# Pulmonary artery sarcoma with mediastinal metastasis: a case report

**DOI:** 10.3389/fonc.2025.1724701

**Published:** 2026-01-12

**Authors:** Jin Cai, Bin Nan, Fangbin Liu, Yinghui Ge, Zhiping Guo, Xiaojing Kan

**Affiliations:** 1 Department of Radiology, Central China Subcenter of National Center for Cardiovascular Diseases, Fuwai Central China Cardiovascular Hospital, Central China Fuwai Hospital of Zhengzhou University, Zhengzhou, China; 2 Department of Radiology, Third Affiliated Hospital of Henan University of Traditional Chinese Medicine, Zhengzhou, China; 3 Henan Provincial Health Management Center, Zhengzhou, China

**Keywords:** dual-energy CT, hemodynamics, mediastinal lymph node metastasis, pulmonary artery aneurysms, pulmonary artery sarcoma

## Abstract

We report a 67-year-old man presenting with a 9-month history of intermittent cough and chest pain. Dual-energy CT (DECT) demonstrated: (1) continuous filling defects extending from the main pulmonary artery through the right pulmonary artery into multiple segmental branches; (2) a middle mediastinal soft tissue mass closely related to the left pulmonary artery, left atrium, and left superior pulmonary vein, with spectral curve slope matching that of the intraluminal lesion in the pulmonary artery; (3) multiple irregular aneurysms in the left lower pulmonary artery; (4) cavitation in the right lung. Pulmonary artery biopsy confirmed undifferentiated pulmonary artery sarcoma. Pulmonary angiography revealed multiple left pulmonary artery aneurysms, and the right lung cavitation improved with antifungal therapy. This case demonstrates that pulmonary artery sarcoma can metastasize to the mediastinum, with metastatic lesions invading arteries and causing aneurysms. DECT plays a crucial role in diagnosis.

## 1 Introduction

Pulmonary artery sarcoma is a rare and highly malignant tumor characterized by nonspecific clinical manifestations, often leading to misdiagnosis as pulmonary embolism. Dual-energy computed tomography (DECT), employing dual-energy acquisition and material decomposition techniques, enables the analysis of differential X-ray attenuation across various tissue types, thereby demonstrating potential advantages in the diagnosis and differential diagnosis of pulmonary artery sarcoma. Tumor-associated pulmonary artery aneurysms are exceedingly rare. This report presents the first documented case of pulmonary artery sarcoma with mediastinal metastasis invading and causing a contralateral pulmonary artery aneurysm, aiming to enhance understanding of the imaging characteristics of this disease entity.

## 2 Case report

A 67-year-old male patient was admitted in December 2023 with a chief complaint of “intermittent cough accompanied by chest pain for 9 months.” The patient reported predominantly dry cough with occasional expectoration of small amounts of white mucoid sputum, without hemoptysis or fever. The chest pain was localized to the left thorax, manifesting as intermittent dull pain unrelated to body position or respiration. The patient had a 20-year smoking history (approximately 30 pack-years) and denied any history of tuberculosis, hypertension, or diabetes mellitus.

Physical examination upon admission revealed: body temperature 36.8°C, pulse 82 beats/min, respiratory rate 18 breaths/min, and blood pressure 128/76 mmHg. Bilateral breath sounds were clear with no audible rales, and cardiopulmonary auscultation demonstrated no abnormalities. Laboratory investigations showed: white blood cell count (WBC) 8.2×10^9^/L, neutrophil percentage (NEU%) 68.5%, hemoglobin (Hb) 132 g/L, and platelet count (PLT) 245×10^9^/L; C-reactive protein (CRP) 18.4 mg/L (reference value <10 mg/L); erythrocyte sedimentation rate (ESR) 35 mm/h (reference value <20 mm/h); D-dimer 4.32 mg/L (reference value <0.55 mg/L); serum (1, 3)-β-D-glucan and bronchoalveolar lavage galactomannan assays were positive; and tumor markers including carcinoembryonic antigen (CEA) 3.5 ng/mL (reference value <5 ng/mL) and squamous cell carcinoma antigen (SCC) 1.2 ng/mL (reference value <1.5 ng/mL), both within normal limits.

**Figure 1:**
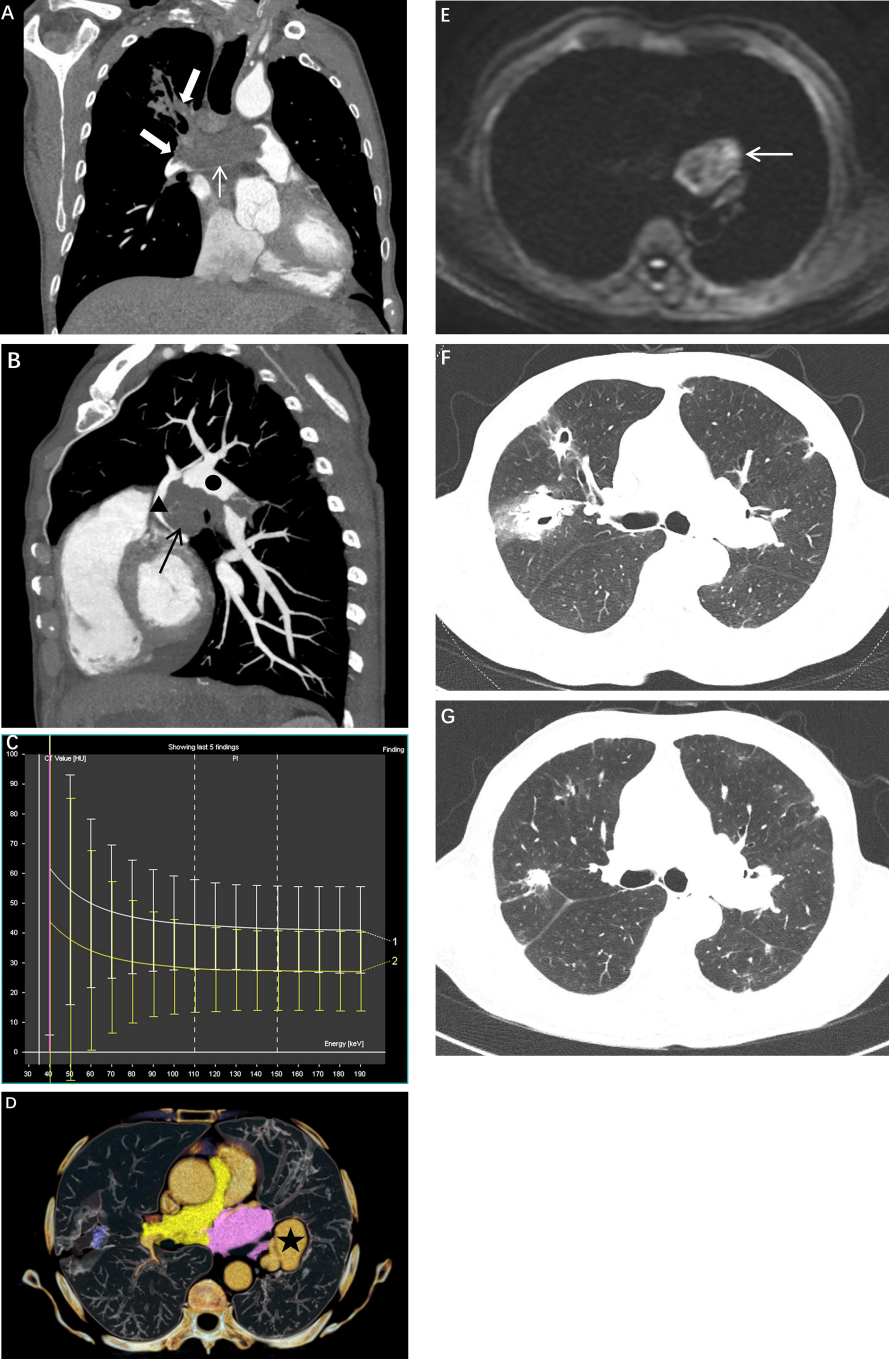
**(A)** Coronal CT image showing filling defects in the main pulmonary artery and right pulmonary artery (white arrows and thick white arrow); **(B)** Sagittal CT image demonstrating the close relationship between the mediastinal mass (black arrow), left pulmonary artery (solid black circle), and left atrium (black triangle); **(C)** Three-dimensional reconstruction displaying a rupture site of the left lower pulmonary artery aneurysm (★); **(D)** Spectral curve showing concordance between the pulmonary artery lesion (white curve) and the mediastinal mass (yellow curve); **(E)** Diffusion-weighted imaging (DWI) revealing the mediastinal mass (white arrow) with heterogeneous hyperintense signal; **(F)** Axial CT image at initial presentation demonstrating cavitation in the right upper lobe; **(G)** Axial CT image obtained 2 months after antifungal therapy showing marked reduction in cavity size.

Dual-energy computed tomography (DECT) imaging demonstrated continuous irregular elongated soft tissue density filling defects within the main pulmonary artery extending to the right pulmonary artery and multiple segmental pulmonary arteries (**Figure 1A**), with CT attenuation values of approximately 25 Hounsfield units (HU) in the arterial phase and 30 HU in the venous phase. On magnetic resonance imaging (MRI), these lesions exhibited prolonged T1 and hyperintense fat-suppressed T2 signals, with predominantly hypointense diffusion-weighted imaging (DWI) signals and no significant enhancement on contrast-enhanced sequences. An oval soft tissue density mass was identified in the middle mediastinum (**Figure 1B**), measuring approximately 55 HU in the arterial phase and 60 HU in the venous phase, demonstrating prolonged T1 and heterogeneous T2 signals with heterogeneously hyperintense DWI signals (**Figure 1E**). Contrast-enhanced imaging revealed mild heterogeneous enhancement, with the mass showing close anatomical relationship to the left pulmonary artery, left atrium, and left superior pulmonary vein; bronchial arterial branches were visualized within the mass. Notably, the dual-energy spectral curves of the middle mediastinal mass and the intraluminal lesion of the main pulmonary artery-right pulmonary artery demonstrated highly concordant slopes (**Figure 1D**), suggesting a common origin. CT revealed multiple irregular aneurysms of the left lower pulmonary artery, with one demonstrating a distinct communication with the pulmonary artery lumen (**Figure 1C**). MRI demonstrated a mass with heterogeneous signal intensity adjacent to the left lower pulmonary artery trunk, exhibiting enhancement consistent with pulmonary arterial enhancement. CT imaging revealed multiple thick-walled cavities and patchy consolidations of varying sizes in both lungs, with surrounding halo signs. The largest lesion was located in the right upper lobe, measuring approximately 2.5 cm in diameter (**Figure 1F**). In combination with the laboratory findings, the patient was diagnosed with possible pulmonary aspergillosis according to the EORTC/MSG criteria. After 2 months of voriconazole treatment, follow-up CT showed significant resolution of the cavitary lesions (**Figure 1G**).

Pulmonary angiography demonstrated a filling defect in the right main pulmonary artery and aneurysmal dilatation of the distal left main pulmonary artery. Late arterial phase imaging revealed aneurysmal dilatation of the left lower pulmonary artery trunk.Intraoperatively, aspiration biopsy of the right pulmonary artery lesion yielded four fragments of tissue. Pulmonary artery pressure was measured at 53 mmH_2_O.

Histopathological examination of the pulmonary artery lesion revealed that the tumor was composed of markedly atypical spindle cells and pleomorphic cells, with frequent pathological mitotic figures.Immunohistochemical staining showed: CD31 (positive in vascular walls), CD34 (−), CK (−), Desmin (−), EMA (−), Factor VIII (positive in vascular walls), Ki-67 (40%+), S-100 (−), SMA (focally positive), CDK4 (+), MDM2 (scattered +), MUC4 (−), MyoD1 (focally positive), P16 (+), SOX10 (−), Vimentin (+), Calponin (scattered positive in vascular walls), and STAT6 (−). Fluorescence *in situ* hybridization (FISH) revealed no MDM2 gene amplification.The pathological diagnosis was undifferentiated sarcoma. The final diagnosis was undifferentiated pulmonary artery sarcoma with mediastinal invasion and pulmonary artery aneurysm formation. The patient declined surgical intervention and chemotherapy; palliative treatment was administered.

## 3 Discussion

Pulmonary artery sarcoma is a rare and highly malignant neoplasm characterized by nonspecific clinical manifestations, typically presenting with dyspnea, chest pain, and cough, frequently resulting in misdiagnosis as pulmonary embolism (1). The present patient presented solely with intermittent cough and chest pain at initial consultation. The atypical clinical presentation coupled with concurrent multiple pulmonary pathologies contributed to diagnostic delay.

The differentiation between pulmonary artery sarcoma (PAS) and pulmonary embolism (PE) is of critical importance, as both entities may manifest as filling defects within the pulmonary arteries on imaging. However, several distinguishing features can aid in their differentiation:(1) PAS predominantly involves the main pulmonary artery and the left and right pulmonary artery trunks, presenting as a contiguous filling defect. The “wall eclipsing sign” may be observed (1, 2), and the proximal tumor margin typically forms an acute angle with the pulmonary artery wall (3). In contrast, PE more commonly affects lobar and segmental pulmonary arteries, presenting as segmental filling defects.(2) PAS demonstrates expansile growth, which may result in dilatation of the distal pulmonary artery and irregular vessel wall contour, whereas the vessel wall in PE typically remains smooth and regular. (3) PAS may exhibit mild or delayed enhancement following contrast administration, whereas PE characteristically shows no enhancement. (4) PE does not result in mediastinal metastasis. In the present case, spectral analysis on dual-energy CT (DECT) demonstrated that the pulmonary artery lesion and mediastinal mass exhibited identical spectral curves, suggesting metastatic spread. This characteristic is valuable for differentiating neoplastic lesions from thrombus.

Conventional CT imaging demonstrates inherent limitations in diagnosing intraluminal pulmonary arterial lesions, particularly in distinguishing tumor emboli from thrombi. DECT, employing dual-energy acquisition and material decomposition techniques, enables analysis of differential X-ray attenuation across various tissue types (4), thereby enhancing diagnostic sensitivity and specificity for pulmonary artery sarcoma. In this case, DECT not only revealed filling defects within the pulmonary artery but also demonstrated, through spectral curve analysis, the potential common origin of the mediastinal mass and pulmonary arterial lesion—a finding of significant value in determining disease nature and extent.

Nevertheless, imaging modalities provide solely morphological and functional information; definitive diagnosis remains dependent on histopathological confirmation. In this case, intrapulmonary arterial biopsy established the diagnosis of undifferentiated pulmonary artery sarcoma, with pathological features demonstrating atypical spindle cells arranged in band-like patterns with prominent pathological mitotic figures. Immunohistochemical markers, such as SMA, CD34, CD99, CD117, S-100, and Desmin, are valuable for differentiating sarcoma subtypes. Pulmonary artery intimal sarcoma (PAIS) represents the most common histological subtype of pulmonary artery sarcoma, and MDM2 gene amplification serves as a key diagnostic criterion for PAIS. Notably, pulmonary artery sarcoma may exhibit histological overlap with other soft tissue sarcomas, including angiosarcoma and undifferentiated pleomorphic sarcoma; therefore, immunohistochemistry and genetic testing are crucial for establishing a definitive diagnosis.

Beyond pulmonary embolism, pulmonary artery sarcoma requires differential diagnosis from other space-occupying lesions, including: (1) angiomatoid fibrous histiocytoma of the pulmonary artery, characterized by tortuous vascular structures visible during the arterial phase; and (2) metastatic tumors involving the pulmonary artery, which typically occur in patients with a history of lung cancer or other malignancies, and the enhancement pattern often resembles that of the primary tumor.

Pulmonary artery aneurysms are predominantly attributed to congenital factors or pulmonary arterial hypertension; tumor-associated pulmonary artery aneurysms are exceedingly rare (5). This case represents the first reported instance of pulmonary artery sarcoma causing contralateral pulmonary artery aneurysm through mediastinal metastatic invasion. The proposed mechanism involves: sarcomatous invasion of the right pulmonary artery resulting in impeded blood flow, subsequently increasing hemodynamic pressure in the left pulmonary artery. Sustained elevated pressure weakens the arterial wall, culminating in aneurysm formation. Literature reports indicate that tumor-associated pseudoaneurysms are more frequently encountered (6), whereas the formation of true aneurysms in this case was closely associated with hemodynamic alterations.

The concurrent pulmonary fungal infection in this case may be attributed to sarcoma-related immunosuppression and localized pulmonary tissue hypoxia secondary to pulmonary artery sarcoma. Comprehensive evaluation of such patients is of particular importance. Elevated D-dimer levels are observed in both malignant neoplasms and pulmonary embolism; in this case, the elevation likely reflects increased thrombotic risk, a factor that warrants consideration in differential diagnosis.

This case underscores that in patients presenting with chronic cough, chest pain, and pulmonary arterial filling defects, pulmonary artery sarcoma should be considered in the differential diagnosis. DECT, as a noninvasive diagnostic modality, can provide substantial support for clinical diagnosis and merits broader clinical application. However, definitive diagnosis remains dependent on histopathological examination, including tissue biopsy, immunohistochemical analysis, and genetic testing. Early diagnosis and intervention are crucial for improving prognosis.

## 4 Conclusion

Pulmonary artery sarcoma may undergo mediastinal metastasis, with metastatic lesions capable of invading the pulmonary artery and inducing aneurysm formation. Additionally, ipsilateral pulmonary fungal infection observed in conjunction with pulmonary artery sarcoma suggests the tumor’s impact on the local microenvironment. DECT demonstrates significant diagnostic value in differential diagnosis. Clinically, in patients presenting with nonspecific symptoms accompanied by pulmonary arterial abnormalities, rare etiologies should be considered. Histopathological examination and molecular biological techniques play pivotal roles in establishing definitive diagnosis and provide essential guidance for therapeutic decision-making.

## Data Availability

The original contributions presented in the study are included in the article/supplementary material. Further inquiries can be directed to the corresponding author.
